# From bad calls to system errors: accountability in automated and assisted sports officiating

**DOI:** 10.3389/fspor.2026.1788299

**Published:** 2026-07-24

**Authors:** Jun Woo Kwon

**Affiliations:** Sports Technology Laboratory, Department of Physical Education, Seoul National University, Seoul, Republic of Korea

**Keywords:** accountability, automated and assisted officiating, decision-making, error, sports technology

## Abstract

Automated and assisted officiating technologies are often introduced in elite sport as ways to improve accuracy, consistency, and fairness. Studies of VAR and Hawk-Eye show that these technologies can improve some decisions, but they also show that technology does not simply remove error from officiating. It can change where error is located, how it is described, and who is expected to answer for it. This article focuses on that shift. The main issue is not only whether automated systems reduce incorrect calls, but also how mistakes are explained when decisions depend on referees, protocols, tracking systems, data, software, and governing bodies at the same time. The article uses the term “error without accountability” for cases in which an error is recognized, but the process for explaining, contesting, correcting, or assigning responsibility for that error remains unclear. This does not mean that responsibility disappears. Rather, responsibility is spread across several human, technical, and institutional actors, making accountability harder to locate. This problem is close to existing debates on algorithmic accountability, responsibility gaps, and AI governance, but the article applies it to the specific setting of automated and assisted sports officiating.

## 1 Introduction

Automated and assisted officiating technologies are now part of elite sport. VAR in football and Hawk-Eye in racket sports are usually justified as ways to reduce human error and make decisions more accurate and consistent. Studies of VAR have shown improvements in several aspects of decision-making, including the correction of some incorrect calls and changes in the decision process itself (1–3). Work on Hawk-Eye and tennis review systems also shows that technology can improve some decisions, although it may also shift the kinds of errors that occur rather than simply remove them (4, 5). In this article, “accuracy” is used in this broader officiating sense, meaning whether a decision is treated as correct under the rules and review procedures of the sport, rather than only as a technical classification measure.

This means that the debate cannot be reduced to whether technology is more accurate than human judgment. Sports technology has long produced controversy as well as improvement, especially when new systems change how decisions are made, challenged, and accepted (6, 7). Recent work on AI and sport also points to problems of transparency, responsibility, and governance when automated systems are used in settings with competitive and legal consequences (8, 9).

The broader literature on algorithmic systems has already examined accountability, opacity, responsibility gaps, and the reviewability of automated decisions (10–15). The claim here is therefore narrower. Sports officiating raises a specific version of the problem because an officiating error is not only an incorrect outcome. It is also something that can be explained, contested, corrected, or assigned to a responsible actor.

The article asks what happens to that role of error when decisions are made through referees, review protocols, tracking systems, software, data, and governing bodies at the same time. It argues that automated and assisted officiating do not remove error from sport. They can change how error is named and where responsibility for it is located. The article uses the term “error without accountability” to describe cases in which an officiating error is recognized, but the process for explaining, contesting, correcting, or assigning responsibility remains unclear.

## 2 Automated, assisted, and hybrid officiating

The distinction between human and automated officiating should not be treated as a simple opposition. Human officiating has never been only a matter of individual judgment. Referees make decisions within rules, review procedures, competition regulations, and institutional expectations about expertise and authority (16). A wrong call may be linked to a particular official, but the conditions under which that call is made, reviewed, and accepted are also shaped by the sport's governing structures.

Automated and assisted officiating systems are also not uniform. VAR is better understood as a decision-support system than as a fully automated referee. It depends on video review, replay operators, communication protocols, and the final authority of the on-field referee (2, 3). Hawk-Eye and related line-calling systems move further toward automation, but their operation still depends on calibration, data capture, system approval, and rules about when the system's output is binding or contestable (4, 5).

For this reason, the issue is not a shift from clear human responsibility to responsibility-free technology. The better description is a shift in how responsibility is distributed. In many contemporary systems, officiating decisions are produced through a hybrid arrangement of referees, technical systems, protocols, governing bodies, and technology providers. This article uses that hybrid setting as the starting point for discussing error and accountability.

## 3 From bad calls to system errors

A bad call and a system error are not just two names for the same problem. They point to different ways of explaining a mistake. A bad call directs attention to the judgment of an official: what the referee saw, how the rule was applied, and whether the decision should have been reviewed or corrected. A system error directs attention elsewhere. It asks whether the camera angle, tracking data, calibration, replay protocol, or software output worked as expected.

This change in language matters because it changes the route through which accountability is pursued. In human officiating, an incorrect decision can usually be discussed as a failure of perception, interpretation, or judgment. That does not mean responsibility is always simple, but the responsible role is visible. In technologically mediated officiating, the same incorrect outcome may be treated as the result of a technical limit, a protocol problem, or a system condition. The question then becomes harder to place: was the error made by the referee, the review process, the data system, the software, the operator, or the governing body that approved the system?

This is why the shift from bad calls to system errors should not be understood as a purely semantic change. It affects how mistakes are challenged and who is expected to explain them. Research on sport technology has shown that new officiating systems can reduce some controversies while producing new ones around implementation, authority, and trust (6, 7). Work on algorithmic opacity and accountability also shows that when decisions are produced through technical systems, it can become difficult to identify how an outcome was generated and who should answer for it (10–12).

The point is not that every technical explanation avoids accountability. Some systems may include clear review procedures and assigned responsibilities. The problem arises when an error is acknowledged but the route for explaining, contesting, or correcting it is unclear. In those cases, the mistake has not disappeared. It has been moved into a sociotechnical process where responsibility is harder to locate.

This can be seen in recent disputes over VAR and goal-line technology. In Tottenham Hotspur vs. Liverpool in 2023, PGMOL released the VAR audio after Luis Díaz's goal was incorrectly disallowed and explained the error through the review process and communication between officials (17). In Aston Villa vs. Sheffield United in 2020, Hawk-Eye apologized after the goal-line system failed to signal a goal because the cameras were occluded (18). These cases are different, but both show how disputed decisions can move from the language of a single bad call to questions about protocol, communication, calibration, data capture, and system operation.

## 4 Error without accountability

The concept of error without accountability is used here in a limited sense. It does not mean that no one is responsible when technology is used in officiating. It also does not mean that automated systems always create a complete gap in responsibility. The problem is narrower. Error without accountability occurs when an officiating error is recognized, but there is no clear process for explaining it, contesting it, correcting it, or assigning responsibility for it.

Responsibility and accountability are related, but they are not the same. Responsibility refers to the roles and duties attached to a decision: who operates the system, who applies the protocol, who approves the technology, and who has the authority to make or revise a call. Accountability refers to the process through which those actors can be asked to explain, justify, or correct what happened. Responsibility may be spread across several actors while accountability procedures remain clear. The problem arises when responsibility is spread across several actors but the route for accountability is unclear.

This argument builds on work on responsibility gaps and algorithmic accountability, but it uses those debates for a more specific purpose. Responsibility gap debates ask who can be held responsible when an outcome is produced through a complex human and technical system, and they also caution against assuming too quickly that technology creates a complete gap in responsibility (13, 14). Algorithmic accountability debates ask how automated systems can be made explainable, justifiable, auditable, reviewable, or open to challenge (10–12, 15). These debates are useful, but they do not fully describe the problem that arises in officiating when an error is recognized but no clear route exists for explaining, contesting, correcting, or assigning responsibility for it.

This is where the concept of error without accountability is useful. It focuses on what happens after an error has already occurred. In human officiating, a bad call can usually be discussed as the judgment of a referee or officiating crew. It can be criticized, reviewed, or corrected through existing procedures. In assisted or automated officiating, the same kind of error may be explained through a review protocol, tracking system, calibration process, software output, or institutional rule. The error may still affect the result of a match, but it becomes less clear who should explain it, how it can be challenged, or whether it can be corrected. The concept therefore helps show how technology can change not only the source of error, but also the process through which error is made accountable.

Table 1 summarizes this argument. The examples are drawn from the preceding discussion of refereeing expertise, VAR, Hawk-Eye, goal-line technology, and sport-technology controversy (2–7, 16–18). The table also shows how accountability mechanisms and forms of contestation change when officiating decisions depend more heavily on review protocols, tracking systems, software outputs, and institutional procedures (11–15).

**Table 1:** Error, accountability, and contestation in automated and assisted officiating.

Officiating setting	Representative example	Where the decision is made	How error is usually described	Main accountability mechanism	Form of contestation
Human officiating	Referee judgment on a foul, penalty, offside, or boundary call	Referee judgment within rules and competition procedures	Bad call, misperception, mistaken rule interpretation	Identification, evaluation, or review of the official who made the decision	Immediate protest, post-match criticism, formal complaint, referee review
Assisted officiating	VAR review in football; Hawk-Eye challenge-based review in tennis	Referee judgment supported by video review, replay operators, or tracking data	Review error, communication problem, protocol failure, interpretive disagreement	Shared responsibility between the on-field official, review officials, and the review protocol	Contestation remains possible, but usually only within predefined review rules and time limits
More automated officiating	Goal-line technology; automated or electronic line-calling systems	System output based on tracking, calibration, data capture, or software processing	System error, calibration issue, occlusion, data limitation, technical failure	Technical validation, system approval, provider explanation, governing-body oversight	Contestation becomes more limited because the output is often treated as authoritative or final
Hybrid sociotechnical officiating	VAR offside decision involving on-field officials, VAR officials, replay review, and communication protocol	Decision produced across referees, protocols, technical systems, governing bodies, and providers	Error remains visible, but its source is distributed across human, technical, and institutional actors	Accountability depends on whether the sport provides clear procedures for explanation, review, correction, and attribution	Contestation shifts from challenging a referee's judgment to challenging a wider sociotechnical decision process

The table does not present a fixed sequence from human judgment to full automation. It shows how the form of error, the location of decision-making authority, and the available routes of contestation change when officiating decisions depend more heavily on review protocols, tracking systems, software outputs, technology providers, and institutional approval. The examples are illustrative rather than exhaustive.

## 5 Governance, trust, and contestation

The governance issue is not that automated and assisted systems sometimes make errors. All officiating systems do. The issue is whether a sport has clear procedures for explaining and contesting errors made through several human and technical actors. If the referee, review protocol, tracking system, operator, governing body, and technology provider all play some role, accountability has to be organized across that arrangement.

Descriptively, this means that officiating authority is no longer located only in the referee's judgment. It is also shaped by review protocols, technical outputs, system approval, and institutional rules about when a decision can be challenged or corrected. When an error occurs, the question is not only whether the decision was right or wrong. It is also how the decision was produced and which actor had authority at each stage.

Normatively, the issue is what procedures should exist when a technologically mediated decision is disputed. This article makes a narrower claim: if routes of explanation and correction are unclear, athletes, teams, and spectators may find it harder to understand how the decision was produced and who can answer for it. Work on algorithmic accountability makes a similar point in other settings: accountability depends on whether decisions can be explained and justified, not only on whether a system performs well (11, 12). AI governance research and work on reviewable automated decision-making add that accountability also requires procedures for review, audit, and challenge after a decision has been made (15, 19).

Accuracy should not be the only criterion for judging officiating technologies. A system may be highly accurate overall and still leave difficult questions after a disputed decision. Who had authority over it? Was there a review process? Could the error be corrected? Was the cause technical, procedural, or interpretive? These questions show why governance matters after accuracy has been improved.

## 6 Discussion and future research

The main value of “error without accountability” is not that it replaces responsibility gaps or algorithmic accountability. It brings those debates closer to sports officiating. Responsibility gap debates explain why it can be difficult to identify a responsible actor. Algorithmic accountability debates explain why explanation, audit, review, and challenge are needed. This article adds that, in sport, an error also has to be understood through the procedures that allow it to be explained, contested, corrected, or attributed.

Future research could examine this problem empirically. One approach would be to compare official statements, review protocols, and appeal procedures after disputed decisions involving VAR, Hawk-Eye, or other tracking systems. Another would be to study how athletes, coaches, referees, and spectators understand responsibility when a decision is produced through both human judgment and technical systems. These questions could also be compared with other settings where decisions are assisted by AI. The sporting context is distinctive because decisions are public, time-sensitive, and tied to competitive outcomes.

As an Opinion article, it does not empirically demonstrate how often error without accountability occurs, or how athletes, officials, or spectators respond to it. It offers a conceptual argument and questions for future empirical work.
